# Synthesis, crystal structure and Hirshfeld surface analysis of Fmoc-β-amino butyric acid and Fmoc carbamate

**DOI:** 10.1107/S2056989025003810

**Published:** 2025-05-13

**Authors:** Mubarak Abubakar Magaji, Beining Chen, Craig Collumbine Robertson

**Affiliations:** a School of Mathematical and Physical Sciences, Dainton Building, University of Sheffield, Brook Hill, Sheffield, S3 7HF, United Kingdom; University of Buenos Aires, Argentina

**Keywords:** crystal structure, Fmoc-β-amino butyric acid and Fmoc carbamate

## Abstract

In the context of the development of synthetic routes that facilitate the incorporation of β-amino acids into peptide synthesis, the synthesis, crystal structure and Hirshfeld surface analysis are reported of fluorenyl­meth­oxy­carbonyl (Fmoc) protected β-amino butyric acid, namely, 3-{[(9*H*-fluoren-9-ylmeth­oxy)carbon­yl]amino}­butanoic acid, C_19_H_19_NO_4_. The importance of pH control in the reaction employing Fmoc-N_3_ is demonstrated with another β-amino acid analogue from which Fmoc carbamate was identified as the major product.

## Abstract 2

In the context of the development of synthetic routes that facilitate the incorporation of β-amino acids into peptide synthesis, the synthesis, crystal structure and Hirshfeld surface analysis are reported of fluorenyl­meth­oxy­carbonyl (Fmoc) protected β-amino butyric acid. The importance of pH control in the reaction employing Fmoc-N_3_ is demonstrated with another β-amino acid analogue from which Fmoc carbamate was identified as the major product.

## 1. Chemical context

The increasing application of non-natural amino acids, particularly β-amino acids, in drug development, specifically peptide drugs necessitates the development of an economical and cost-effective method for producing modified β-amino acids. Modern peptide synthesis predominantly employs solid-phase peptide synthesis (SPPS) using the Fmoc strategy to mask the reactivity of the amine group with a temporary protective group (Behrendt *et al.*, 2016 ▸). Peptide chain elongation can then be performed through sequential cycles involving the removal of the protective group, followed by the coupling of *N*-protected amino acids (Hlebowicz *et al.*, 2005 ▸; Isidro-Llobet *et al.*, 2007 ▸). This strategy allows efficient and controlled peptide assembly. Fmoc chemistry, while seemingly straightforward, presents challenges, particularly with β-amino acids due to the additional α-carbon, which serves as a potential reactive site. During the optimization of the synthesis of Fmoc-β-amino acids using alternatives to Fmoc-Cl, Fmoc-*R*-β-amino­butyric acid (Fmoc-*R*-βABA) **1** was synthesized to a high yield and purity after the *in situ* preparation of Fmoc-N_3_ (Cruz *et al.*, 2004 ▸). When the same technique was applied to the synthesis of Fmoc-dl-β-phenyl­alanine, Fmoc-carbamate **2** was obtained. 
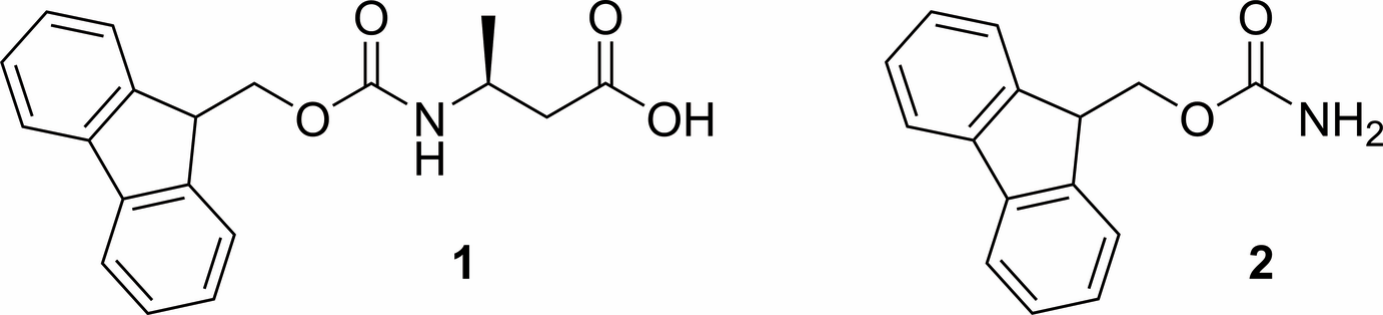


## 2. Structural commentary

**Figure 1:**
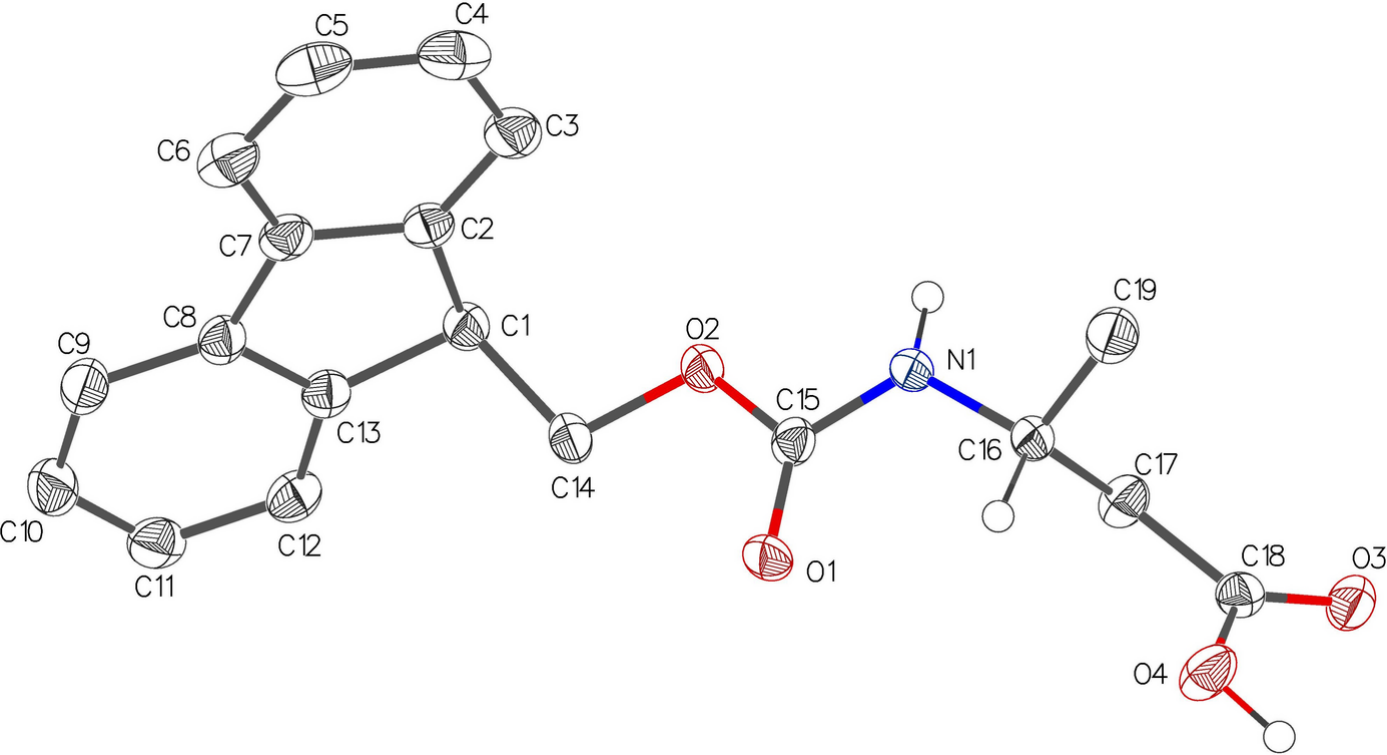
The mol­ecular structure of Fmoc-β-amino butyric acid **1** showing 50% displacement ellipsoids

**Figure 2:**
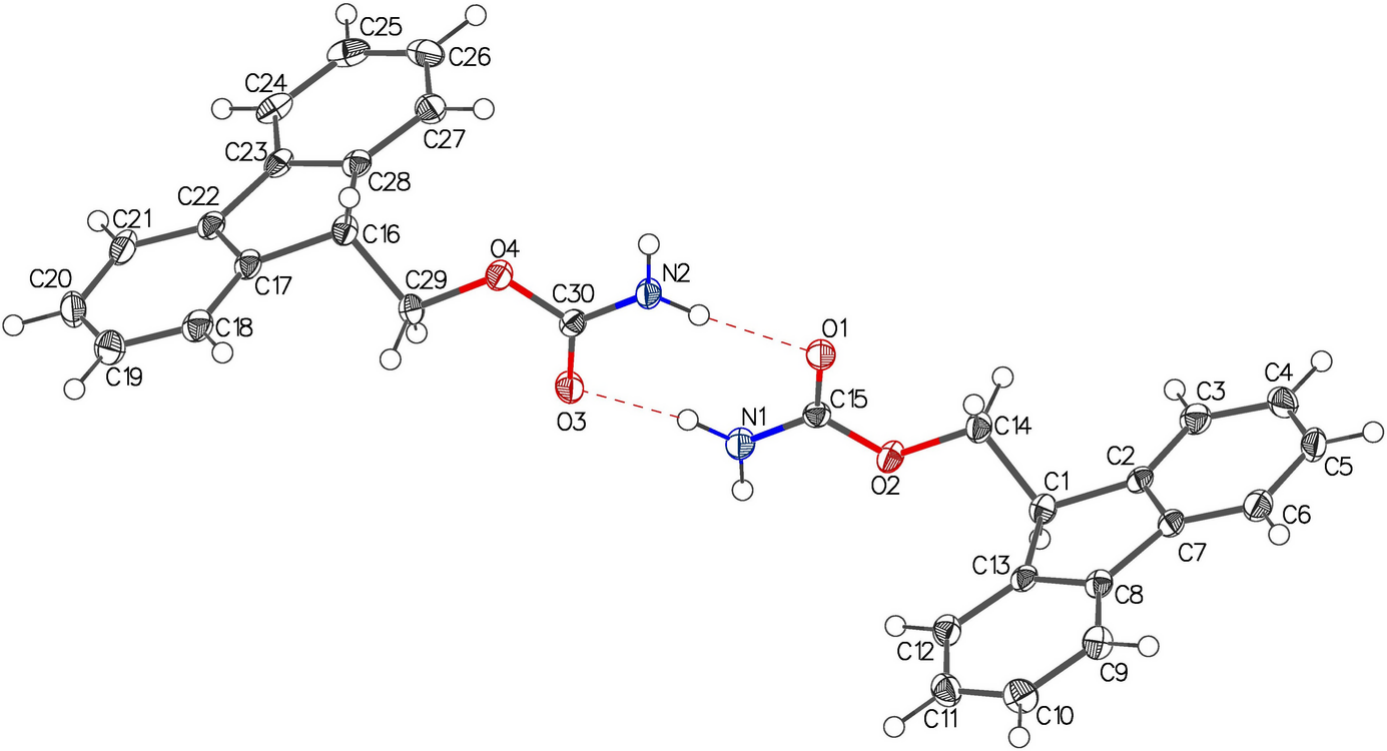
The mol­ecular structure of Fmoc carbamate **2** showing 50% displacement ellipsoids

Compound **1** crystallizes in the ortho­rhom­bic space group *P*2_1_2_1_2_1_, its asymmetric unit comprising of a single mol­ecule (Fig. 1 ▸). The tricyclic fluorenyl group is planar (r.m.s. deviation 0.025 Å). The carbamate group adopts the *trans* geometry and is planar (r.m.s. deviation 0.005 Å). The absolute configuration of an *R* stereogenic centre is confirmed to a high degree of certainty. The Hooft parameter of −0.05 (14) demonstrates that a single enanti­omer is present. Compound **2** crystallizes in the ortho­rhom­bic space group *Pca*2_1_, its asymmetric unit comprising of two mol­ecules (Fig. 2 ▸). The two tricyclic fluorenyl groups are both planar (r.m.s. deviation of C16–C28 = 0.020 Å and C1–C13 = 0.025 Å). The carbamate group is planar (r.m.s. deviation 0.001 Å). The absolute configuration of an *R* stereogenic centre is confirmed to a high degree of certainty. The Hooft parameter of −0.05 (14) demonstrates that a single enanti­omer is present.

## 3. Supra­molecular features

**Figure 3:**
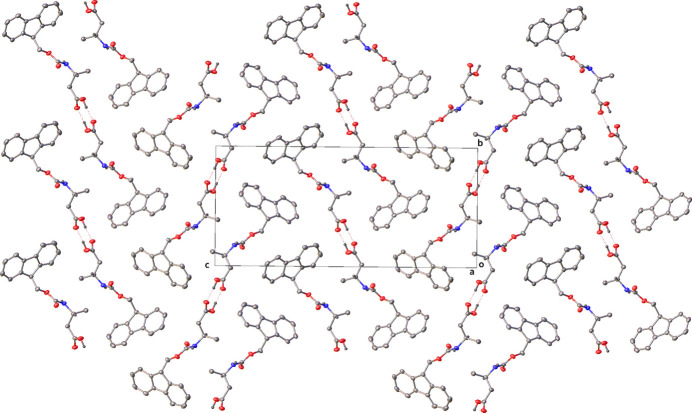
Packing of **1** as viewed along the *a* axis

**Figure 4:**
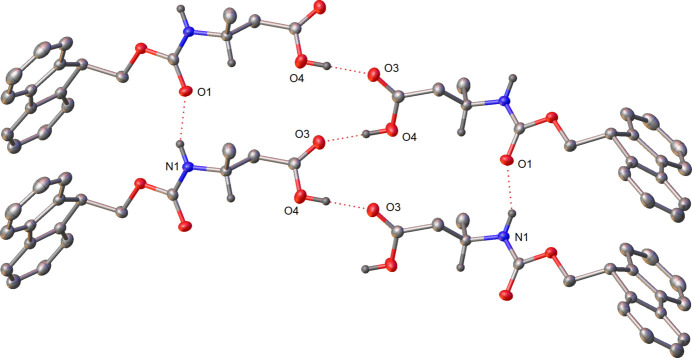
Hydrogen bonding present in **1**

In the mol­ecular packing of crystal **1** (Fig. 3 ▸), two hydrogen-bonded (Table 1 ▸) chains are observed (Fig. 4 ▸). One chain forms from the carbamate hydrogen atom (H1) and the carbonyl oxygen atom (O1) of an adjacent mol­ecule (−1 + *x*, *y*, *z*) and continues parallel to the *a* axis (Fig. 4 ▸). The second chain observed is formed by the carb­oxy­lic acid group hydrogen atom (H4) and the carbonyl oxygen atom (O3) of the adjacent mol­ecule (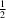 + *x*, 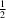 − *y*, 1 − *z*). These chains are not linear but have an angle of 47.9 (10)° between the carb­oxy­lic acid planes of each mol­ecule. Hydrogen-bond statistical analysis (*Mercury* 2024.1.0; Macrae *et al.*, 2020 ▸) was performed, which highlighted the hydrogen bonds to be not unusual. The transamide chain hydrogen bond [2.844 (2) Å] is a little below average in distance (2.97 Å) from 1428 hits. The carb­oxy­lic acid chain hydrogen bond [2.656 (2) Å] was found to be of average distance (2.66 Å) from 3072 hits.

**Figure 5:**
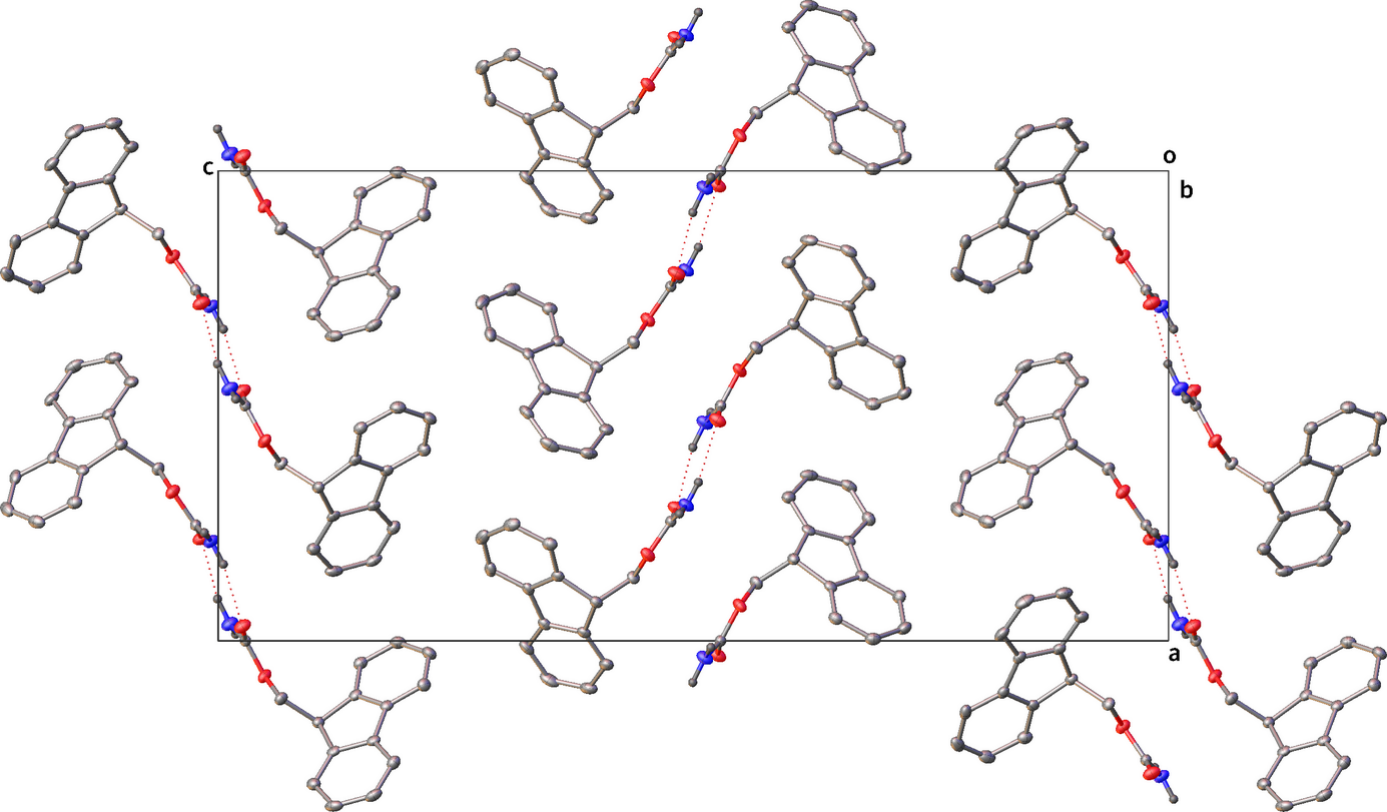
Packing of **2** as viewed along the *b* axis

**Figure 6:**
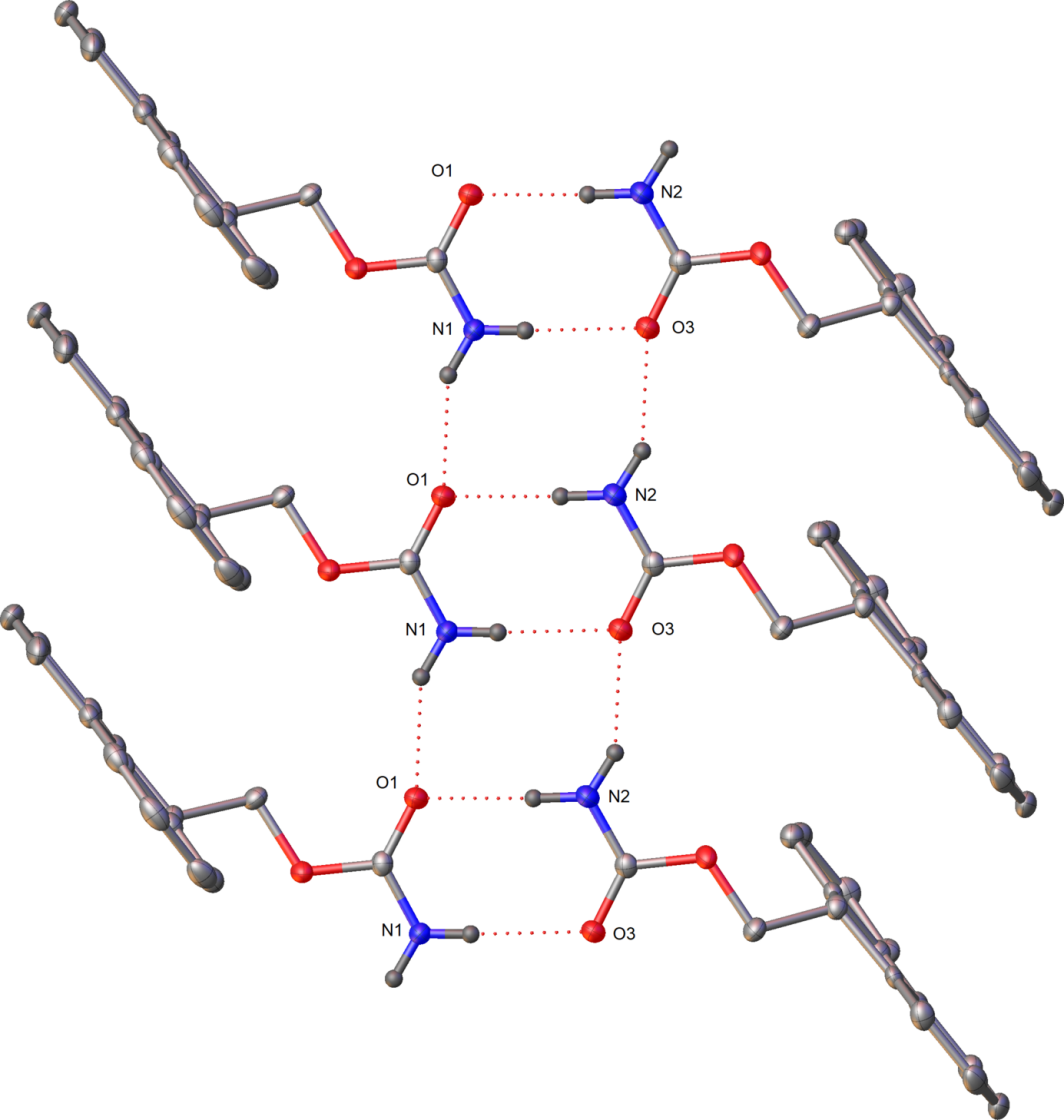
Hydrogen bonding between the carbamate groups present in **2**

**Figure 7:**
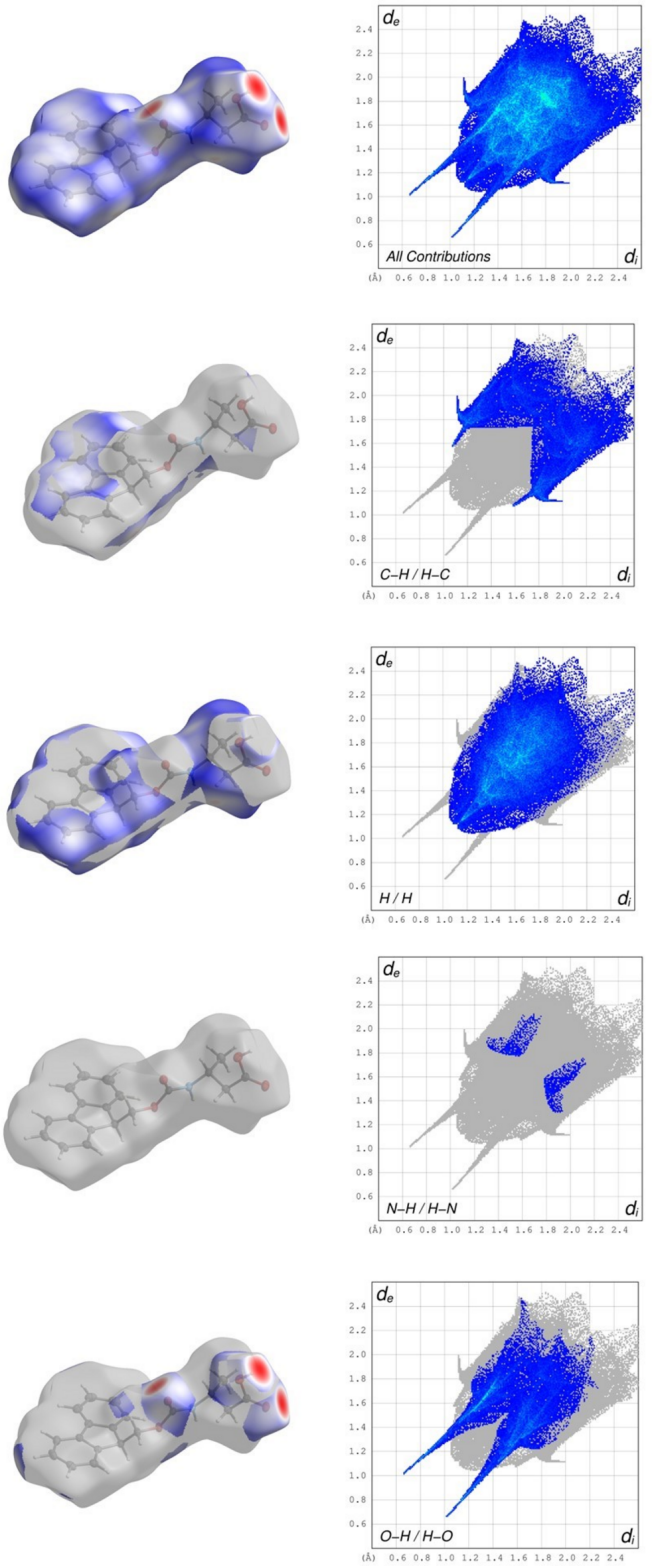
Hirshfeld surfaces of **1** mapped with *d*_norm_ (left image of each pair) with the corresponding two-dimensional fingerprint plot (right image of each pair) showing firstly all contributions and then the major contributions of C⋯H/H⋯C, H⋯H, N⋯H/H⋯N and O⋯H/H⋯O contacts.

**Figure 8:**
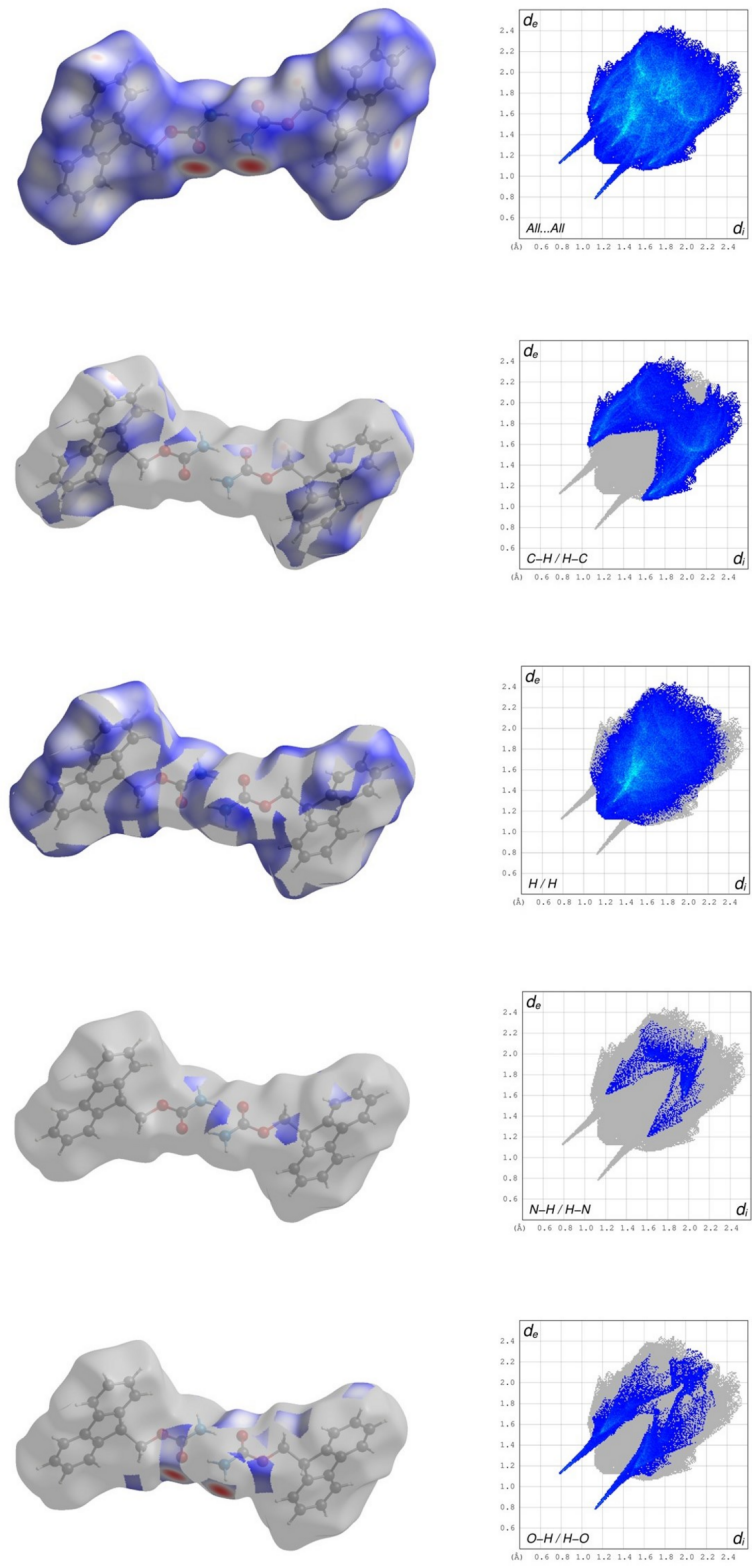
Hirshfeld surfaces of **2** mapped with *d*_norm_ (left image of each pair) with the corresponding two-dimensional fingerprint plot (right image of each pair) showing firstly all contributions and then the major contributions of C⋯H/H⋯C, H⋯H, N⋯H/H⋯N and O⋯H/H⋯O contacts.

Strong face–face π–π interactions (as investigated with the CSD Materials Aromatics Analyser tool) are observed in the packing of **1**, running parallel to the *a* axis. The rings C1–C6 and C8–13 form columns stacking on top and below with symmetry equivalents (*x*, *y*, *z* and −1 + *x*, *y*, *z*), with a centroid–centroid distance of 4.8393 (2) Å and twist plane angle of 0.0 (3)°. These interactions can be visualised later in the Hirshfield surface analysis plot (Fig. 7, 2nd from top) showing the C⋯H/H⋯C interactions primarily around the Fmoc group.In the mol­ecular packing of crystal **2** (Fig. 5 ▸), the two mol­ecules in the asymmetric unit dimerize with hydrogen bonds (Table 2 ▸) formed between the carbamate hydrogen atom (H2*A*) and oxygen atom (O1), as well as the carbamate hydrogen atom (H1*B*) and oxygen atom (O3). The other carbamate hydrogen atoms of each of the two mol­ecules then form a further hydrogen bond to an adjacent mol­ecule to form a 1D hydrogen-bonded network (Fig. 6 ▸). The carbamate hydrogen atom (H1*A*) forms a hydrogen bond with oxygen atom (O1) (*x*, −1 + *y*, *z*) and the carbamate hydrogen atom (H2*B*) with oxygen atom (O3) (*x*, 1 + *y*, *z*). Hydrogen-bond statistical analysis (*Mercury* 2024.1.0; Macrae *et al.*, 2020 ▸) was performed, which allowed comparison to 3268 structures. This showed that the dimeric hydrogen bonds (N2—H2*A*⋯O1) and (N1—H1*B*⋯O3) were not unusual. The hydrogen bonds (N1—H1*A*⋯O1) and (N2—H2*B*⋯O3) were found to be unusual on account of their hydrogen bonds having shorter lengths and more acute angles [2.8549 (3) Å, 149 (3)° and 2.827 (3) Å, 152 (3)°, respectively] below the mean (2.94 Å and 173.35°). A strong face–face π–π interaction is observed in the packing of **2** between the rings C17–C22 and C23–-28 (*x*, −1 + *y*, *z*) as well as between C2–C7 and C8–C13(*x*, 1 + *y*, *z*) columns stacking on top and below with symmetry equivalents), with a centroid–centroid distance of 4.4182 (15) Å and relative orientation of 2.55 (9)°. These interactions can also be visualised later in the Hirshfield surface analysis plot (Fig. 8, 2nd from top) showing the ⋯H/H⋯C interactions around the Fmoc group.

## 4. Database survey

A search of the Cambridge Structural Database (CSD version 5.46, November 2024; Groom *et al.*, 2016 ▸) highlighted that the crystal structures of compounds **1** and **2** have not been reported. Searches for structural motifs similar to **1** began with Fmoc-protected α-amino acids and discovered 51 results of various natural amino acids, synthetic derivatives and co-crystals, the most closely related structure being CUWKIO (Valle *et al.*, 1984 ▸), a Fmoc-protected alanine monohydrate that differs in structure by the one carbon atom as well as co-crystallizing with a mol­ecule of water. Despite crystallizing in the same space group as **1**, CUWKIO has a different set of hydrogen bonds formed within the crystal structure. Instead of the amide hydrogen-bonded chains seen in **1**, the amide H atom of CUWKIO forms a hydrogen bond to the carb­oxy­lic acid carbonyl whereas the co-crystallized water forms a hydrogen bond to the amide carbonyl. The co-crystallized water then satisfies its remaining hydrogen-bond formation with the carb­oxy­lic acid carbonyl and the carb­oxy­lic acid hydrogen is satisfied by forming a hydrogen bond to the water. In total, four hydrogen bonds are reported in CUWKIO in contrast to **1**, which has two. Aromatic inter­actions, as investigated with the CSD Materials Aromatics Analyser tool, highlight a change in packing of the π–π inter­actions of the Fmoc groups. Strong face-face inter­actions in **1** are no longer present but instead strong edge-to-face inter­actions seen in CUWKIO. Further investigation of structural similarities lead us to look for Fmoc-protected β-amino acids, the structural motif of which was found the related structure BOMRAY (Ahmad Wani *et al.*, 2014 ▸), which differs as there is a spiro-centred carbon with cyclic six-membered ring at the β carbon, in contrast to **1**, which has the methyl group as well as being a racemate. BOMRAY crystallises in the *P*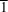 space group and the primary hydrogen-bond inter­actions are a not unusual dimerization of the carb­oxy­lic acid as well as a long amide N—H to carb­oxy­lic acid carbonyl bond. The amide carbonyl in this case only forms a long weak inter­molecular inter­action with a CH_2_ group. Analysis of the aromatic inter­actions in BOMRAY reveals strong face-face π-stacking inter­actions, albeit with an offset, meaning only one of the phenyl rings is involved in the inter­action.

## 5. Hirshfeld surface analysis

In order to visualise the inter­molecular inter­actions in **1** and **2**, Hirshfeld surface analysis was carried out using *CrystalExplorer* 21.5 (Spackman *et al.*, 2021 ▸) and visualised *via* two-dimensional fingerprint plots (McKinnon *et al.*, 2007 ▸). The Hirshfeld surface analysis of **2** was carried out with the asymmetric unit of the two mol­ecules. The left columns of Fig. 7 ▸ (top) and 8 (top) show the Hirshfeld surfaces of **1** and **2**, respectively, each mapped with the function *d*_norm_, which is the sum of the distances from a surface point to the nearest inter­ior (*d*_i_) and exterior(*d*_e_) atom, normalized by the van der Waals (vdW) radii of the corresponding atom (r_vdW_). Contacts shorter than the sum of their vdW radii are shown in red, those longer in blue and those approximately equal to their vdW radii in white. In the structure of **1**, Fig. 7 ▸, the shortest contacts, with the most intense red spots, are shown to be the hydrogen-bonding sites, as shown in Fig. 4 ▸, the carb­oxy­lic acid and amide. The fingerprint plots (Tan *et al.*, 2019 ▸) for **1** and **2** are given in the right columns of Figs. 7 ▸ and 8 ▸ and the inter­molecular inter­actions shown in Tables 3 ▸ and 4 ▸, respectively. The overall fingerprint plots are shown first (top) followed by those delineated into C⋯H/H⋯C, H/H, N⋯H/H⋯N and O⋯H/H⋯O. For **1**, the most important overall contribution is H⋯H, Fig. 7 ▸, contributing 51.8% with the tip of *d*_e_ = *d*_i_ at 1.12 Å. The shortest inter­actions, the hydrogen bonding at the carb­oxy­lic acid and amide sites, as highlighted by the *d*_norm_ surface plot, are clearly visualized in the bottom O⋯H/H⋯O plot with the shortest distances. The C⋯H⋯π inter­actions are shown in the the surface of the C⋯H/H⋯C highlighted figure as well as the characteristic wings revealed in the fingerprint plot. For structure **2**, while the dimerization of the amine and carbonyl within the asymmetric unit is occluded from the view, the surface map shows the brightest red spots corresponding to the hydrogen bond formed from the amine to carbonyl, clearly highlighted in Fig. 8 ▸ (bottom) surface displaying O⋯H/H⋯O contacts and its corresponding plot demonstrating the shortest distance. In **2**, the most important contribution again is H⋯H, contributing 51.9% with the tip of *d*_e_ = *d*_i_ at 1.16 Å.

## 6. Synthesis and crystallization

Fmoc-Cl was initially derivatized into Fmoc-N_3_ to prepare it for addition to solutions of the β-amino acid. Specifically, 10 mmol of Fmoc-Cl were dissolved in dioxane (5 ml), while 12 mmol of NaN_3_ were dissolved in a 2:1 mixture of dioxane/water (10 ml). The Fmoc-Cl solution was then added to the NaN_3_ solution, and the resulting mixture was stirred at 323 K for 2 h.>

**Figure 9:**
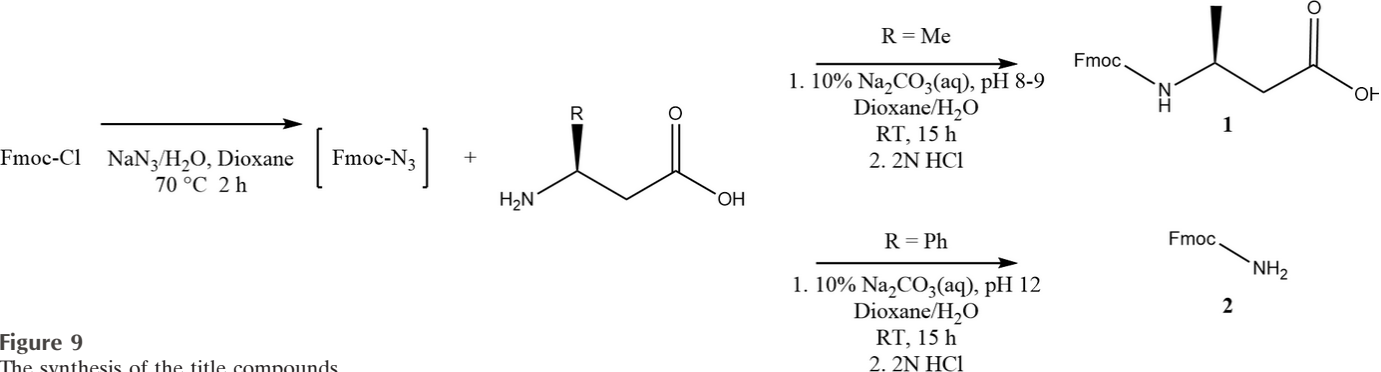
The synthesis of the title compounds.

For the synthesis of **1**, (Fig. 9 ▸)11 mmol of R-βABA were dissolved in a 2:1 mixture of dioxane/10% NaHCO_3_, which maintains the pH at 8–9. The Fmoc-N_3_ solution was cautiously added in three portions to the β-amino acid over a period of 1 h. The reaction mixture was then stirred for 15 h at room temperature. Following the reaction, the mixture was poured into 5 mL of ice-cold water and subjected to three extractions with petroleum ether. The aqueous layers were separated using a separation funnel and chilled on ice for 2 h. Subsequently, the aqueous layer was acidified to pH 1 with 2 *M* HCl. The resulting precipitate was filtered and washed with ice-cold water until a pH of about 5 was attained. The collected white solid was placed in a petri dish covered with a paper towel and left to dry overnight within the fume hood. X-ray quality single crystals were grown by recrystallization from ethyl acetate/pet ether. **NMR**: ^1^H NMR (400 MHz, DMSO) δ = 12.18 (*s*, 1H), 7.89 (*dd*, *J* = 7.3, 5.9, 3H), 7.73–7.62 (*m*, 3H), 7.47–7.26 (*m*, 7H), 4.63 (*d*, *J* = 6.2, 1H), 4.38–4.17 (*m*, 3H), 3.88 (*hept*, *J* = 6.8, 1H), 2.50–2.42 (*m*, 1H), 2.30 (*dd*, *J* = 15.4, 7.3, 1H), 1.10 (*d*, *J* = 6.6, 2H). ^13^C NMR (101 MHz, DMSO) δ 128.03, 127.32, 125.56, 125.21, 120.29, 69.65, 65.78, 65.43, 47.14, 47.14, 46.44, 44.33, 41.51, 41.51, 41.16, 40.11, 21.12, 20.77. MP:120-125 °C HRMS Analysis: *m*/*z* (ES^+^) 326.1401. C_19_H_20_NO_4_ requires 326.1387.

For the synthesis of **2**, 11 mmol of dl-β-phenyl­alanine were dissolved in a 2:1 mixture of dioxane/10% NaHCO_3_ along with NH_4_OH (1 mL), which maintains the pH at 12. As above, the Fmoc-N_3_ solution was cautiously added in three portions to the β-amino acid over a period of 1 h. The reaction mixture was then stirred for 15 h at room temperature. Following the reaction, the mixture was poured into 5 mL of ice-cold water and subjected to three extractions with petroleum ether. The aqueous layers were separated using a separation funnel and chilled on ice for 2 h. Subsequently, the aqueous layer was acidified to pH 1 with 2 *M* HCl. The resulting precipitate was filtered and washed with ice-cold water until a pH of about 5 was attained. The collected white solid was placed in a Petri dish covered with a paper towel and left to dry overnight within the fume hood. X-ray quality single crystals were grown by recrystallization from ethyl acetate/pet ether. M.p. 471–473 K NMR: ^1^H NMR (400 MHz, DMSO) δ = 7.89 (*d*, *J* = 7.5, 2H), 7.70 (*d*, *J* = 7.4, 2H), 7.46–7.38 (*m*, 2H), 7.34 (*td*, *J* = 7.4, 1.2, 2H), 6.75 (*s*, 1H), 6.55 (*s*, 1H), 4.28 (*d*, *J* = 1.6, 1H), 4.27 (*s*, 1H), 4.22 (*dd*, *J* = 8.0, 5.7, 1H). ^13^C NMR (101 MHz, DMSO) δ = 157.20, 157.13, 144.45, 143.05, 141.21, 139.90, 137.90, 129.39, 128.06, 127.76, 127.52, 125.62, 121.85, 120.57, 120.49, 110.19, 65.48, 47.22. HRMS Analysis: *m*/*z* (ES^+^) C_15_H_13_NO_2_ requires 239.26.

## 7. Refinement

Crystal data, data collection and structure refinement details are summarized in Table 5 ▸. All carbon-bound H atoms were positioned geometrically and refined as riding, with aromatic C—H = 0.95 Å, *sp*^3^ C—H = 1.00 Å, *sp*^3^ C—H_2_ 0.99 Å and with *U*_iso_(H) = 1.2 *U*_eq_(C) and *sp*^3^ C—H_3_ = 0.98 Å with *U*_iso_(H) = 1.5*U*_eq_(methyl C). Hydrogen atoms involved in hydrogen-bonding inter­actions were refined isotropically.

## Supplementary Material

Crystal structure: contains datablock(s) 1, 2. DOI: 10.1107/S2056989025003810/vu2009sup1.cif

Structure factors: contains datablock(s) 1. DOI: 10.1107/S2056989025003810/vu20091sup2.hkl

Supporting information file. DOI: 10.1107/S2056989025003810/vu20091sup4.cml

Structure factors: contains datablock(s) 2. DOI: 10.1107/S2056989025003810/vu20092sup3.hkl

CCDC references: 2447425, 2447424

Additional supporting information:  crystallographic information; 3D view; checkCIF report

## Figures and Tables

**Table 1:** Hydrogen-bond geometry (Å, °) for **1**[Chem scheme1]

*D*—H⋯*A*	*D*—H	H⋯*A*	*D*⋯*A*	*D*—H⋯*A*
O4—H4⋯O3^i^	0.94 (4)	1.72 (4)	2.656 (2)	177 (3)
N1—H1⋯O1^ii^	0.88 (3)	2.04 (3)	2.844 (3)	152 (2)

Symmetry codes: (i) 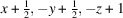; (ii) 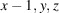.

**Table 2:** Hydrogen-bond geometry (Å, °) for **2**[Chem scheme1]

*D*—H⋯*A*	*D*—H	H⋯*A*	*D*⋯*A*	*D*—H⋯*A*
N1—H1*B*⋯O3	0.91 (3)	2.06 (3)	2.955 (3)	169 (3)
N1—H1*A*⋯O1^i^	0.86 (3)	2.08 (3)	2.849 (3)	149 (3)
N2—H2*A*⋯O1	0.85 (3)	2.13 (3)	2.967 (3)	169 (3)
N2—H2*B*⋯O3^ii^	0.87 (4)	2.03 (4)	2.827 (3)	152 (3)

Symmetry codes: (i) 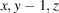; (ii) 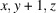.

**Table 3:** Summary of the percentages of inter­molecular contacts contributed to the HSA surface of Fmoc-protected β-amino­butyric acid **1**

Inside atom		Outside atom			Total contributions
	C	H	N	O	
C	2.0	13.0	0.0	0.2	15.3
H	9.8	51.8	0.5	9.8	71.9
N	0.0	0.5	0.0	0.0	0.5
O	0.2	11.6	0.0	0.5	12.3
Total contributions	12.0	77.0	0.5	10.5	

**Table 4:** Summary of the percentages of inter­molecular contacts contributed to the HSA surface of Fmoc carbamate **2**

Inside atom		Outside atom			Total contributions
	C	H	N	O	
C	2.6	17.9	0.0	0.1	20.7
H	13.6	51.9	1.2	4.9	71.6
N	0.0	1.2	0.0	0.2	1.4
O	0.1	5.5	0.1	0.6	6.4
Total contributions	16.3	76.6	1.3	5.8	

**Table 5:** Experimental details

	**1**	**2**
Crystal data		
Chemical formula	C_19_H_19_NO_4_	C_15_H_13_NO_2_
*M*_r_	325.35	239.26
Crystal system, space group	Orthorhombic, *P*2_1_2_1_2_1_	Orthorhombic, *Pca*2_1_
Temperature (K)	100	100
*a*, *b*, *c* (Å)	4.8393 (2), 12.4928 (4), 27.3101 (9)	15.3560 (3), 5.0400 (1), 31.0254 (7)
*V* (Å^3^)	1651.07 (10)	2401.19 (9)
*Z*	4	8
Radiation type	Cu *K*α	Cu *K*α
μ (mm^−1^)	0.75	0.71
Crystal size (mm)	0.3 × 0.03 × 0.01	0.25 × 0.03 × 0.01

Data collection		
Diffractometer	Bruker D8 Venture Photon III	Bruker D8 Venture Photon III
Absorption correction	Multi-scan (*SADABS*; Krause *et al.*, 2015 ▸)	Multi-scan (*SADABS*; Krause *et al.*, 2015 ▸)
*T*_min_, *T*_max_	0.664, 0.753	0.683, 0.753
No. of measured, independent and observed [*I* > 2σ(*I*)] reflections	25780, 2922, 2590	18874, 4069, 3754
*R*_int_	0.081	0.051
(sin θ/λ)_max_ (Å^−1^)	0.596	0.596

Refinement		
*R*[*F*^2^ > 2σ(*F*^2^)], *wR*(*F*^2^), *S*	0.032, 0.073, 1.06	0.030, 0.068, 1.05
No. of reflections	2922	4069
No. of parameters	226	341
No. of restraints	0	1
H-atom treatment	H atoms treated by a mixture of independent and constrained refinement	H atoms treated by a mixture of independent and constrained refinement
Δρ_max_, Δρ_min_ (e Å^−3^)	0.12, −0.17	0.14, −0.17
Absolute structure	Flack *x* determined using 992 quotients [(*I*^+^)−(*I*^−^)]/[(*I*^+^)+(*I*^−^)] (Parsons *et al.*, 2013 ▸)	Flack *x* determined using 1617 quotients [(*I*^+^)−(*I*^−^)]/[(*I*^+^)+(*I*^−^)] (Parsons et al., 2013 ▸)
Absolute structure parameter	−0.05 (14)	−0.26 (13)

Computer programs: *APEX5* and *SAINT* V8.40B (Bruker, 2023 ▸), *SHELXT2018/2* (Sheldrick, 2015*a* ▸), *SHELXL* (Sheldrick, 2015*b* ▸) and *OLEX2* (Dolomanov *et al.*, 2009 ▸).

## supplementary crystallographic information

### 3-{[(9H-Fluoren-9-ylmethoxy)carbonyl]amino}butanoic acid (1) Crystal data

**Table:**

C_19_H_19_NO_4_	*D*_x_ = 1.309 Mg m^−^^3^
*M**_r_* = 325.35	Cu *K*α radiation, λ = 1.54178 Å
Orthorhombic, *P*2_1_2_1_2_1_	Cell parameters from 3582 reflections
*a* = 4.8393 (2) Å	θ = 3.2–66.6°
*b* = 12.4928 (4) Å	µ = 0.75 mm^−^^1^
*c* = 27.3101 (9) Å	*T* = 100 K
*V* = 1651.07 (10) Å^3^	Needle, colourless
*Z* = 4	0.3 × 0.03 × 0.01 mm
*F*(000) = 688	

### 3-{[(9H-Fluoren-9-ylmethoxy)carbonyl]amino}butanoic acid (1) Data collection

**Table:**

Bruker D8 Venture Photon III diffractometer	2590 reflections with *I* > 2σ(*I*)
φ and ω scans	*R*_int_ = 0.081
Absorption correction: multi-scan (SADABS; Krause *et al.*, 2015)	θ_max_ = 66.8°, θ_min_ = 3.2°
*T*_min_ = 0.664, *T*_max_ = 0.753	*h* = −5→5
25780 measured reflections	*k* = −14→14
2922 independent reflections	*l* = −32→31

### 3-{[(9H-Fluoren-9-ylmethoxy)carbonyl]amino}butanoic acid (1) Refinement

**Table:**

Refinement on *F*^2^	Hydrogen site location: mixed
Least-squares matrix: full	H atoms treated by a mixture of independent and constrained refinement
*R*[*F*^2^ > 2σ(*F*^2^)] = 0.032	*w* = 1/[σ^2^(*F*_o_^2^) + (0.0304*P*)^2^ + 0.2044*P*] where *P* = (*F*_o_^2^ + 2*F*_c_^2^)/3
*wR*(*F*^2^) = 0.073	(Δ/σ)_max_ < 0.001
*S* = 1.06	Δρ_max_ = 0.12 e Å^−^^3^
2922 reflections	Δρ_min_ = −0.17 e Å^−^^3^
226 parameters	Absolute structure: Flack *x* determined using 992 quotients [(*I*^+^)-(*I*^-^)]/[(*I*^+^)+(*I*^-^)] (Parsons *et al.*, 2013)
0 restraints	Absolute structure parameter: −0.05 (14)
Primary atom site location: dual	

### 3-{[(9H-Fluoren-9-ylmethoxy)carbonyl]amino}butanoic acid (1) Special details

**Table:**

Geometry. All esds (except the esd in the dihedral angle between two l.s. planes) are estimated using the full covariance matrix. The cell esds are taken into account individually in the estimation of esds in distances, angles and torsion angles; correlations between esds in cell parameters are only used when they are defined by crystal symmetry. An approximate (isotropic) treatment of cell esds is used for estimating esds involving l.s. planes.
Refinement. For 1, a needle crystal with dimensions 0.01 x 0.026 x 0.3 mm was selected and for 2, a needle crystal with dimensions 0.012 x 0.026 x 0.25 was selected. Intensity data for each was collected on a Bruker Venture Photon III diffractometer operating with a CuKα microfocus X-ray source with the crystal mounted in fomblin oil on a MicroMount (MiTeGen, USA) and cooled to 100 K in a stream of cold nitrogen gas using an Oxford Cryosystems 700 Cryostream. Data were corrected for absorption using empirical methods (SADABS; Bruker, 2023) based upon symmetry equivalent reflections combined with measurements at different azimuthal angles (Krause *et al.*, 2015). The crystal structures were solved and refined against F^2^ values using ShelXT (Sheldrick, 2015a) for solution and ShelXL (Sheldrick, 2015b) for refinement accessed *via* the Olex2 program (Dolomanov *et al.*, 2009). Non-hydrogen atoms were refined anisotropically.

### 3-{[(9H-Fluoren-9-ylmethoxy)carbonyl]amino}butanoic acid (1) Fractional atomic coordinates and isotropic or equivalent isotropic displacement parameters (Å^2^)

**Table:**

	*x*	*y*	*z*	*U*_iso_*/*U*_eq_	
O1	0.6114 (3)	0.65660 (13)	0.60381 (6)	0.0260 (4)	
O2	0.2958 (3)	0.76326 (12)	0.64093 (6)	0.0254 (4)	
O3	−0.0146 (4)	0.30716 (13)	0.53156 (6)	0.0309 (4)	
O4	0.3887 (4)	0.38097 (14)	0.51041 (7)	0.0331 (4)	
H4	0.422 (8)	0.314 (3)	0.4965 (14)	0.078 (12)*	
N1	0.1735 (4)	0.67252 (15)	0.57425 (7)	0.0215 (4)	
H1	0.005 (6)	0.691 (2)	0.5827 (9)	0.032 (7)*	
C1	0.4148 (5)	0.89471 (18)	0.70131 (8)	0.0230 (5)	
H1A	0.227847	0.885997	0.716402	0.028*	
C2	0.4183 (5)	0.99043 (18)	0.66703 (8)	0.0231 (5)	
C3	0.2681 (5)	1.0088 (2)	0.62449 (9)	0.0309 (6)	
H3	0.133824	0.958606	0.613666	0.037*	
C4	0.3181 (6)	1.1019 (2)	0.59806 (10)	0.0372 (7)	
H4A	0.216012	1.115393	0.568994	0.045*	
C5	0.5141 (6)	1.1755 (2)	0.61339 (10)	0.0374 (7)	
H5	0.546508	1.238106	0.594522	0.045*	
C6	0.6638 (6)	1.15843 (19)	0.65610 (10)	0.0326 (6)	
H6	0.797817	1.208928	0.666704	0.039*	
C7	0.6136 (5)	1.06620 (19)	0.68294 (9)	0.0250 (5)	
C8	0.7377 (5)	1.02758 (18)	0.72875 (9)	0.0239 (5)	
C9	0.9348 (5)	1.0736 (2)	0.75932 (10)	0.0304 (6)	
H9	1.010016	1.142041	0.751993	0.037*	
C10	1.0195 (6)	1.0182 (2)	0.80057 (9)	0.0346 (6)	
H10	1.151438	1.049315	0.822029	0.042*	
C11	0.9134 (6)	0.9177 (2)	0.81081 (9)	0.0322 (6)	
H11	0.977890	0.879868	0.838730	0.039*	
C12	0.7136 (5)	0.8712 (2)	0.78084 (9)	0.0273 (6)	
H12	0.639459	0.802678	0.788335	0.033*	
C13	0.6254 (5)	0.92702 (18)	0.73987 (8)	0.0229 (5)	
C14	0.5056 (5)	0.79107 (19)	0.67675 (9)	0.0249 (5)	
H14A	0.686512	0.801056	0.660463	0.030*	
H14B	0.523900	0.733348	0.701367	0.030*	
C15	0.3773 (5)	0.69342 (17)	0.60606 (9)	0.0213 (5)	
C16	0.2032 (5)	0.58643 (17)	0.53839 (8)	0.0212 (5)	
H16	0.404379	0.576644	0.531365	0.025*	
C17	0.0920 (5)	0.48249 (18)	0.56036 (9)	0.0255 (5)	
H17A	0.172825	0.473954	0.593435	0.031*	
H17B	−0.110285	0.490126	0.564482	0.031*	
C18	0.1454 (5)	0.38223 (18)	0.53224 (9)	0.0230 (5)	
C19	0.0586 (6)	0.6161 (2)	0.49085 (9)	0.0314 (6)	
H19A	−0.139338	0.625852	0.497011	0.047*	
H19B	0.084998	0.558750	0.466822	0.047*	
H19C	0.136876	0.682848	0.478078	0.047*	

### 3-{[(9H-Fluoren-9-ylmethoxy)carbonyl]amino}butanoic acid (1) Atomic displacement parameters (Å^2^)

**Table:**

	*U*^11^	*U*^22^	*U*^33^	*U*^12^	*U*^13^	*U*^23^
O1	0.0159 (8)	0.0299 (9)	0.0323 (9)	0.0033 (7)	−0.0010 (7)	−0.0065 (8)
O2	0.0174 (8)	0.0265 (8)	0.0322 (9)	0.0008 (7)	−0.0031 (7)	−0.0098 (7)
O3	0.0281 (9)	0.0220 (8)	0.0427 (11)	−0.0025 (7)	0.0001 (8)	−0.0051 (8)
O4	0.0280 (10)	0.0227 (9)	0.0486 (12)	0.0002 (8)	0.0117 (9)	−0.0072 (8)
N1	0.0147 (10)	0.0212 (10)	0.0287 (11)	0.0010 (8)	−0.0011 (9)	−0.0044 (9)
C1	0.0195 (12)	0.0233 (12)	0.0263 (13)	−0.0004 (10)	−0.0001 (10)	−0.0031 (10)
C2	0.0202 (12)	0.0246 (12)	0.0246 (12)	0.0042 (10)	0.0031 (10)	−0.0036 (10)
C3	0.0278 (14)	0.0346 (14)	0.0304 (13)	0.0074 (12)	−0.0007 (11)	−0.0066 (12)
C4	0.0404 (16)	0.0419 (16)	0.0292 (14)	0.0167 (14)	−0.0002 (12)	0.0046 (12)
C5	0.0420 (16)	0.0315 (15)	0.0385 (16)	0.0098 (13)	0.0110 (13)	0.0094 (12)
C6	0.0316 (15)	0.0262 (13)	0.0402 (15)	0.0026 (12)	0.0074 (12)	0.0010 (12)
C7	0.0225 (12)	0.0235 (12)	0.0290 (13)	0.0040 (10)	0.0045 (10)	−0.0028 (10)
C8	0.0212 (12)	0.0236 (12)	0.0267 (13)	0.0007 (10)	0.0029 (10)	−0.0059 (10)
C9	0.0253 (14)	0.0266 (13)	0.0394 (15)	−0.0021 (11)	0.0021 (11)	−0.0098 (12)
C10	0.0307 (14)	0.0397 (16)	0.0336 (15)	0.0050 (12)	−0.0056 (12)	−0.0159 (13)
C11	0.0351 (15)	0.0360 (14)	0.0254 (13)	0.0116 (12)	−0.0032 (11)	−0.0072 (11)
C12	0.0311 (14)	0.0249 (12)	0.0258 (13)	0.0037 (11)	0.0039 (11)	−0.0036 (10)
C13	0.0204 (12)	0.0239 (12)	0.0243 (12)	0.0014 (10)	0.0038 (10)	−0.0044 (10)
C14	0.0199 (12)	0.0268 (13)	0.0279 (13)	−0.0010 (10)	−0.0042 (10)	−0.0063 (11)
C15	0.0209 (12)	0.0179 (11)	0.0251 (12)	−0.0031 (10)	0.0011 (10)	−0.0005 (10)
C16	0.0175 (11)	0.0203 (11)	0.0258 (12)	−0.0001 (9)	−0.0011 (10)	−0.0032 (10)
C17	0.0269 (12)	0.0220 (12)	0.0276 (13)	−0.0005 (10)	0.0043 (10)	−0.0026 (10)
C18	0.0222 (13)	0.0206 (11)	0.0261 (13)	0.0003 (10)	−0.0025 (10)	0.0025 (10)
C19	0.0382 (16)	0.0261 (13)	0.0300 (14)	0.0002 (12)	−0.0060 (12)	−0.0010 (11)

### 3-{[(9H-Fluoren-9-ylmethoxy)carbonyl]amino}butanoic acid (1) Geometric parameters (Å, º)

**Table:**

O1—C15	1.224 (3)	C7—C8	1.469 (3)
O2—C14	1.452 (3)	C8—C9	1.392 (3)
O2—C15	1.350 (3)	C8—C13	1.402 (3)
O3—C18	1.216 (3)	C9—H9	0.9500
O4—H4	0.94 (4)	C9—C10	1.385 (4)
O4—C18	1.320 (3)	C10—H10	0.9500
N1—H1	0.88 (3)	C10—C11	1.384 (4)
N1—C15	1.340 (3)	C11—H11	0.9500
N1—C16	1.462 (3)	C11—C12	1.394 (4)
C1—H1A	1.0000	C12—H12	0.9500
C1—C2	1.519 (3)	C12—C13	1.386 (3)
C1—C13	1.520 (3)	C14—H14A	0.9900
C1—C14	1.523 (3)	C14—H14B	0.9900
C2—C3	1.389 (3)	C16—H16	1.0000
C2—C7	1.407 (3)	C16—C17	1.528 (3)
C3—H3	0.9500	C16—C19	1.521 (3)
C3—C4	1.391 (4)	C17—H17A	0.9900
C4—H4A	0.9500	C17—H17B	0.9900
C4—C5	1.385 (4)	C17—C18	1.492 (3)
C5—H5	0.9500	C19—H19A	0.9800
C5—C6	1.390 (4)	C19—H19B	0.9800
C6—H6	0.9500	C19—H19C	0.9800
C6—C7	1.387 (3)		
			
C15—O2—C14	115.19 (17)	C10—C11—C12	121.1 (2)
C18—O4—H4	110 (2)	C12—C11—H11	119.4
C15—N1—H1	117.4 (18)	C11—C12—H12	120.7
C15—N1—C16	120.36 (19)	C13—C12—C11	118.5 (2)
C16—N1—H1	117.6 (18)	C13—C12—H12	120.7
C2—C1—H1A	110.5	C8—C13—C1	110.4 (2)
C2—C1—C13	102.15 (19)	C12—C13—C1	129.2 (2)
C2—C1—C14	113.24 (19)	C12—C13—C8	120.4 (2)
C13—C1—H1A	110.5	O2—C14—C1	107.37 (19)
C13—C1—C14	109.72 (19)	O2—C14—H14A	110.2
C14—C1—H1A	110.5	O2—C14—H14B	110.2
C3—C2—C1	129.7 (2)	C1—C14—H14A	110.2
C3—C2—C7	119.9 (2)	C1—C14—H14B	110.2
C7—C2—C1	110.3 (2)	H14A—C14—H14B	108.5
C2—C3—H3	120.6	O1—C15—O2	123.3 (2)
C2—C3—C4	118.8 (3)	O1—C15—N1	125.1 (2)
C4—C3—H3	120.6	N1—C15—O2	111.6 (2)
C3—C4—H4A	119.4	N1—C16—H16	108.3
C5—C4—C3	121.1 (3)	N1—C16—C17	109.11 (19)
C5—C4—H4A	119.4	N1—C16—C19	110.34 (18)
C4—C5—H5	119.7	C17—C16—H16	108.3
C4—C5—C6	120.6 (3)	C19—C16—H16	108.3
C6—C5—H5	119.7	C19—C16—C17	112.4 (2)
C5—C6—H6	120.7	C16—C17—H17A	108.1
C7—C6—C5	118.7 (3)	C16—C17—H17B	108.1
C7—C6—H6	120.7	H17A—C17—H17B	107.3
C2—C7—C8	108.5 (2)	C18—C17—C16	116.76 (19)
C6—C7—C2	120.9 (2)	C18—C17—H17A	108.1
C6—C7—C8	130.6 (2)	C18—C17—H17B	108.1
C9—C8—C7	130.9 (2)	O3—C18—O4	123.5 (2)
C9—C8—C13	120.4 (2)	O3—C18—C17	123.0 (2)
C13—C8—C7	108.7 (2)	O4—C18—C17	113.4 (2)
C8—C9—H9	120.5	C16—C19—H19A	109.5
C10—C9—C8	118.9 (2)	C16—C19—H19B	109.5
C10—C9—H9	120.5	C16—C19—H19C	109.5
C9—C10—H10	119.7	H19A—C19—H19B	109.5
C11—C10—C9	120.5 (2)	H19A—C19—H19C	109.5
C11—C10—H10	119.7	H19B—C19—H19C	109.5
C10—C11—H11	119.4		
			
N1—C16—C17—C18	−170.6 (2)	C9—C8—C13—C12	−1.4 (3)
C1—C2—C3—C4	−177.1 (2)	C9—C10—C11—C12	−1.9 (4)
C1—C2—C7—C6	176.9 (2)	C10—C11—C12—C13	1.0 (4)
C1—C2—C7—C8	−2.4 (3)	C11—C12—C13—C1	179.4 (2)
C2—C1—C13—C8	−0.7 (2)	C11—C12—C13—C8	0.7 (3)
C2—C1—C13—C12	−179.5 (2)	C13—C1—C2—C3	−179.9 (2)
C2—C1—C14—O2	−67.4 (3)	C13—C1—C2—C7	1.9 (2)
C2—C3—C4—C5	0.2 (4)	C13—C1—C14—O2	179.18 (18)
C2—C7—C8—C9	−178.4 (2)	C13—C8—C9—C10	0.5 (4)
C2—C7—C8—C13	1.9 (3)	C14—O2—C15—O1	−1.0 (3)
C3—C2—C7—C6	−1.5 (4)	C14—O2—C15—N1	−179.49 (19)
C3—C2—C7—C8	179.2 (2)	C14—C1—C2—C3	62.2 (3)
C3—C4—C5—C6	−0.8 (4)	C14—C1—C2—C7	−116.0 (2)
C4—C5—C6—C7	0.2 (4)	C14—C1—C13—C8	119.7 (2)
C5—C6—C7—C2	0.9 (4)	C14—C1—C13—C12	−59.1 (3)
C5—C6—C7—C8	−179.9 (2)	C15—O2—C14—C1	161.10 (18)
C6—C7—C8—C9	2.3 (4)	C15—N1—C16—C17	90.3 (3)
C6—C7—C8—C13	−177.3 (3)	C15—N1—C16—C19	−145.8 (2)
C7—C2—C3—C4	0.9 (4)	C16—N1—C15—O1	11.4 (4)
C7—C8—C9—C10	−179.0 (2)	C16—N1—C15—O2	−170.19 (18)
C7—C8—C13—C1	−0.7 (3)	C16—C17—C18—O3	−147.0 (2)
C7—C8—C13—C12	178.2 (2)	C16—C17—C18—O4	36.3 (3)
C8—C9—C10—C11	1.1 (4)	C19—C16—C17—C18	66.7 (3)
C9—C8—C13—C1	179.6 (2)		

### 3-{[(9H-Fluoren-9-ylmethoxy)carbonyl]amino}butanoic acid (1) Hydrogen-bond geometry (Å, º)

**Table:**

*D*—H···*A*	*D*—H	H···*A*	*D*···*A*	*D*—H···*A*
O4—H4···O3^i^	0.94 (4)	1.72 (4)	2.656 (2)	177 (3)
N1—H1···O1^ii^	0.88 (3)	2.04 (3)	2.844 (3)	152 (2)

Symmetry codes: (i) *x*+1/2, −*y*+1/2, −*z*+1; (ii) *x*−1, *y*, *z*.

### (2) Crystal data

**Table:**

C_15_H_13_NO_2_	*D*_x_ = 1.324 Mg m^−^^3^
*M**_r_* = 239.26	Cu *K*α radiation, λ = 1.54178 Å
Orthorhombic, *P**c**a*2_1_	Cell parameters from 6701 reflections
*a* = 15.3560 (3) Å	θ = 3.2–65.9°
*b* = 5.0400 (1) Å	µ = 0.71 mm^−^^1^
*c* = 31.0254 (7) Å	*T* = 100 K
*V* = 2401.19 (9) Å^3^	Needle, colourless
*Z* = 8	0.25 × 0.03 × 0.01 mm
*F*(000) = 1008	

### (2) Data collection

**Table:**

Bruker D8 Venture Photon III diffractometer	3754 reflections with *I* > 2σ(*I*)
φ and ω scans	*R*_int_ = 0.051
Absorption correction: multi-scan (SADABS; Krause *et al.*, 2015)	θ_max_ = 66.8°, θ_min_ = 2.9°
*T*_min_ = 0.683, *T*_max_ = 0.753	*h* = −18→18
18874 measured reflections	*k* = −5→5
4069 independent reflections	*l* = −36→36

### (2) Refinement

**Table:**

Refinement on *F*^2^	Hydrogen site location: mixed
Least-squares matrix: full	H atoms treated by a mixture of independent and constrained refinement
*R*[*F*^2^ > 2σ(*F*^2^)] = 0.030	*w* = 1/[σ^2^(*F*_o_^2^) + (0.0291*P*)^2^ + 0.3089*P*] where *P* = (*F*_o_^2^ + 2*F*_c_^2^)/3
*wR*(*F*^2^) = 0.068	(Δ/σ)_max_ < 0.001
*S* = 1.05	Δρ_max_ = 0.14 e Å^−^^3^
4069 reflections	Δρ_min_ = −0.16 e Å^−^^3^
341 parameters	Absolute structure: Flack *x* determined using 1617 quotients[(*I*^+^)-(*I*^-^)]/[(*I*^+^)+(*I*^-^)] (Parsons et al., 2013)
1 restraint	Absolute structure parameter: −0.26 (13)
Primary atom site location: dual	

### (2) Special details

**Table:**

Geometry. All esds (except the esd in the dihedral angle between two l.s. planes) are estimated using the full covariance matrix. The cell esds are taken into account individually in the estimation of esds in distances, angles and torsion angles; correlations between esds in cell parameters are only used when they are defined by crystal symmetry. An approximate (isotropic) treatment of cell esds is used for estimating esds involving l.s. planes.

### (2) Fractional atomic coordinates and isotropic or equivalent isotropic displacement parameters (Å^2^)

**Table:**

	*x*	*y*	*z*	*U*_iso_*/*U*_eq_	
O1	0.53087 (12)	0.7151 (3)	0.47451 (7)	0.0233 (4)	
O2	0.42477 (11)	0.4384 (3)	0.45115 (6)	0.0198 (4)	
N1	0.53609 (17)	0.2741 (4)	0.48787 (8)	0.0230 (5)	
H1A	0.5141 (19)	0.123 (6)	0.4816 (10)	0.019 (7)*	
H1B	0.589 (2)	0.295 (6)	0.5007 (11)	0.031 (8)*	
C1	0.32636 (17)	0.5633 (5)	0.39449 (9)	0.0176 (6)	
H1	0.293372	0.398198	0.401756	0.021*	
C2	0.26475 (15)	0.7768 (5)	0.37850 (9)	0.0176 (5)	
C3	0.19497 (17)	0.8955 (5)	0.39955 (9)	0.0226 (6)	
H3	0.179526	0.844026	0.427999	0.027*	
C4	0.14832 (17)	1.0913 (6)	0.37805 (11)	0.0263 (6)	
H4	0.100259	1.173510	0.391963	0.032*	
C5	0.17085 (18)	1.1689 (5)	0.33649 (9)	0.0248 (6)	
H5	0.138392	1.303942	0.322415	0.030*	
C6	0.24063 (19)	1.0499 (5)	0.31541 (10)	0.0221 (6)	
H6	0.256156	1.102689	0.287037	0.027*	
C7	0.28718 (16)	0.8528 (5)	0.33653 (9)	0.0176 (5)	
C8	0.36178 (15)	0.6916 (5)	0.32254 (8)	0.0171 (5)	
C9	0.40625 (17)	0.6884 (5)	0.28345 (9)	0.0212 (6)	
H9	0.391122	0.808222	0.261028	0.025*	
C10	0.4733 (2)	0.5061 (5)	0.27793 (11)	0.0251 (7)	
H10	0.503783	0.499597	0.251306	0.030*	
C11	0.49616 (17)	0.3337 (5)	0.31090 (10)	0.0237 (6)	
H11	0.542294	0.210925	0.306624	0.028*	
C12	0.45228 (17)	0.3385 (5)	0.35021 (9)	0.0205 (6)	
H12	0.468555	0.221720	0.372863	0.025*	
C13	0.38445 (19)	0.5168 (5)	0.35564 (10)	0.0170 (6)	
C14	0.37677 (17)	0.6619 (5)	0.43372 (9)	0.0194 (5)	
H14A	0.417409	0.805151	0.425237	0.023*	
H14B	0.336087	0.732822	0.455632	0.023*	
C15	0.50003 (18)	0.4899 (5)	0.47137 (9)	0.0165 (6)	
O3	0.71790 (12)	0.3289 (3)	0.51774 (6)	0.0235 (4)	
O4	0.81702 (12)	0.6052 (3)	0.54788 (6)	0.0203 (4)	
N2	0.71138 (16)	0.7720 (4)	0.50730 (8)	0.0201 (5)	
H2A	0.663 (2)	0.759 (5)	0.4946 (10)	0.019 (7)*	
H2B	0.730 (2)	0.928 (7)	0.5147 (11)	0.033 (9)*	
C16	0.91776 (18)	0.4654 (5)	0.60216 (9)	0.0182 (6)	
H16	0.948852	0.635510	0.595949	0.022*	
C17	0.98232 (16)	0.2503 (5)	0.61506 (9)	0.0187 (5)	
C18	1.05075 (16)	0.1434 (5)	0.59156 (9)	0.0229 (6)	
H18	1.062974	0.204782	0.563253	0.028*	
C19	1.10103 (18)	−0.0548 (6)	0.61018 (11)	0.0282 (7)	
H19	1.148311	−0.128562	0.594474	0.034*	
C20	1.08324 (19)	−0.1464 (5)	0.65126 (11)	0.0300 (7)	
H20	1.118190	−0.283160	0.663310	0.036*	
C21	1.0147 (2)	−0.0408 (5)	0.67526 (10)	0.0248 (7)	
H21	1.002632	−0.103184	0.703529	0.030*	
C22	0.96431 (16)	0.1588 (5)	0.65662 (9)	0.0187 (5)	
C23	0.88995 (16)	0.3076 (5)	0.67375 (8)	0.0183 (5)	
C24	0.84808 (18)	0.2916 (6)	0.71340 (9)	0.0232 (6)	
H24	0.865756	0.164739	0.734293	0.028*	
C25	0.7798 (2)	0.4652 (6)	0.72182 (11)	0.0274 (7)	
H25	0.751130	0.458980	0.748942	0.033*	
C26	0.75307 (19)	0.6472 (5)	0.69102 (10)	0.0266 (6)	
H26	0.706041	0.763484	0.697254	0.032*	
C27	0.79414 (17)	0.6619 (5)	0.65113 (9)	0.0225 (6)	
H27	0.775450	0.786606	0.630113	0.027*	
C28	0.86269 (19)	0.4917 (5)	0.64261 (10)	0.0178 (6)	
C29	0.86563 (17)	0.3768 (5)	0.56314 (9)	0.0186 (5)	
H29A	0.905150	0.311282	0.540284	0.022*	
H29B	0.825305	0.231754	0.571180	0.022*	
C30	0.74632 (18)	0.5541 (5)	0.52380 (9)	0.0164 (5)	

### (2) Atomic displacement parameters (Å^2^)

**Table:**

	*U*^11^	*U*^22^	*U*^33^	*U*^12^	*U*^13^	*U*^23^
O1	0.0227 (10)	0.0147 (9)	0.0326 (11)	−0.0011 (7)	−0.0087 (8)	−0.0015 (8)
O2	0.0215 (10)	0.0167 (9)	0.0211 (10)	−0.0041 (7)	−0.0075 (8)	0.0043 (7)
N1	0.0222 (13)	0.0150 (12)	0.0318 (14)	−0.0029 (9)	−0.0085 (11)	0.0005 (10)
C1	0.0160 (14)	0.0192 (13)	0.0176 (14)	−0.0019 (11)	−0.0010 (11)	0.0023 (10)
C2	0.0145 (11)	0.0163 (12)	0.0220 (14)	−0.0035 (9)	−0.0055 (10)	−0.0027 (11)
C3	0.0177 (13)	0.0271 (14)	0.0231 (15)	−0.0014 (11)	−0.0005 (11)	−0.0039 (11)
C4	0.0146 (13)	0.0256 (14)	0.0388 (18)	0.0021 (11)	−0.0022 (12)	−0.0103 (14)
C5	0.0194 (13)	0.0216 (13)	0.0334 (16)	0.0018 (10)	−0.0108 (11)	−0.0008 (11)
C6	0.0236 (15)	0.0216 (13)	0.0212 (15)	−0.0006 (11)	−0.0047 (12)	0.0012 (11)
C7	0.0135 (12)	0.0169 (13)	0.0225 (14)	−0.0027 (9)	−0.0037 (10)	−0.0020 (10)
C8	0.0145 (12)	0.0159 (13)	0.0208 (14)	−0.0041 (10)	−0.0024 (10)	0.0004 (10)
C9	0.0209 (13)	0.0214 (13)	0.0213 (14)	−0.0013 (11)	0.0002 (11)	0.0037 (10)
C10	0.0197 (16)	0.0308 (16)	0.0248 (18)	−0.0006 (10)	0.0063 (13)	−0.0013 (12)
C11	0.0180 (13)	0.0226 (14)	0.0304 (16)	0.0019 (11)	0.0001 (11)	−0.0014 (11)
C12	0.0192 (13)	0.0186 (13)	0.0235 (15)	−0.0011 (10)	−0.0038 (11)	0.0018 (11)
C13	0.0157 (15)	0.0160 (13)	0.0194 (15)	−0.0034 (9)	−0.0047 (12)	−0.0014 (10)
C14	0.0200 (13)	0.0183 (13)	0.0199 (14)	0.0007 (9)	−0.0044 (11)	0.0033 (11)
C15	0.0160 (13)	0.0188 (13)	0.0149 (14)	0.0002 (9)	0.0005 (11)	−0.0020 (10)
O3	0.0211 (10)	0.0142 (9)	0.0351 (12)	−0.0008 (7)	−0.0083 (8)	−0.0002 (8)
O4	0.0226 (10)	0.0144 (9)	0.0239 (10)	−0.0017 (7)	−0.0079 (8)	0.0020 (8)
N2	0.0195 (12)	0.0145 (11)	0.0263 (13)	−0.0004 (9)	−0.0079 (10)	0.0012 (9)
C16	0.0163 (12)	0.0185 (13)	0.0198 (15)	−0.0012 (9)	−0.0030 (11)	0.0020 (11)
C17	0.0150 (11)	0.0190 (12)	0.0223 (14)	−0.0031 (9)	−0.0043 (10)	−0.0002 (10)
C18	0.0163 (12)	0.0278 (14)	0.0248 (15)	−0.0030 (10)	−0.0019 (11)	−0.0032 (11)
C19	0.0163 (14)	0.0311 (15)	0.0372 (18)	0.0008 (11)	−0.0036 (12)	−0.0071 (13)
C20	0.0256 (14)	0.0213 (14)	0.0432 (19)	0.0054 (11)	−0.0156 (14)	−0.0015 (12)
C21	0.0239 (16)	0.0251 (14)	0.0255 (16)	−0.0028 (11)	−0.0117 (13)	0.0028 (12)
C22	0.0183 (13)	0.0172 (12)	0.0205 (14)	−0.0033 (9)	−0.0056 (11)	−0.0008 (10)
C23	0.0183 (12)	0.0190 (13)	0.0176 (14)	−0.0053 (10)	−0.0044 (10)	0.0000 (10)
C24	0.0260 (14)	0.0268 (15)	0.0167 (13)	−0.0093 (11)	−0.0030 (10)	−0.0014 (11)
C25	0.0258 (17)	0.0327 (16)	0.0236 (18)	−0.0113 (12)	0.0054 (14)	−0.0104 (12)
C26	0.0208 (13)	0.0251 (15)	0.0340 (17)	−0.0021 (11)	0.0041 (12)	−0.0124 (12)
C27	0.0197 (13)	0.0202 (13)	0.0275 (15)	−0.0009 (10)	−0.0020 (12)	−0.0033 (11)
C28	0.0147 (14)	0.0186 (14)	0.0201 (16)	−0.0043 (9)	−0.0015 (12)	−0.0022 (10)
C29	0.0202 (13)	0.0166 (13)	0.0189 (13)	0.0030 (10)	−0.0025 (10)	0.0028 (10)
C30	0.0153 (13)	0.0197 (13)	0.0142 (14)	−0.0006 (10)	−0.0003 (11)	−0.0004 (11)

### (2) Geometric parameters (Å, º)

**Table:**

O1—C15	1.234 (3)	O3—C30	1.230 (3)
O2—C14	1.450 (3)	O4—C29	1.451 (3)
O2—C15	1.340 (3)	O4—C30	1.343 (3)
N1—H1A	0.86 (3)	N2—H2A	0.85 (3)
N1—H1B	0.91 (3)	N2—H2B	0.87 (4)
N1—C15	1.323 (4)	N2—C30	1.325 (3)
C1—H1	1.0000	C16—H16	1.0000
C1—C2	1.516 (4)	C16—C17	1.523 (4)
C1—C13	1.518 (4)	C16—C28	1.519 (4)
C1—C14	1.526 (4)	C16—C29	1.518 (4)
C2—C3	1.390 (4)	C17—C18	1.388 (4)
C2—C7	1.400 (4)	C17—C22	1.397 (4)
C3—H3	0.9500	C18—H18	0.9500
C3—C4	1.390 (4)	C18—C19	1.389 (4)
C4—H4	0.9500	C19—H19	0.9500
C4—C5	1.391 (4)	C19—C20	1.383 (5)
C5—H5	0.9500	C20—H20	0.9500
C5—C6	1.391 (4)	C20—C21	1.395 (4)
C6—H6	0.9500	C21—H21	0.9500
C6—C7	1.388 (4)	C21—C22	1.395 (4)
C7—C8	1.470 (4)	C22—C23	1.466 (4)
C8—C9	1.392 (4)	C23—C24	1.390 (4)
C8—C13	1.397 (4)	C23—C28	1.403 (4)
C9—H9	0.9500	C24—H24	0.9500
C9—C10	1.391 (4)	C24—C25	1.391 (4)
C10—H10	0.9500	C25—H25	0.9500
C10—C11	1.387 (4)	C25—C26	1.387 (5)
C11—H11	0.9500	C26—H26	0.9500
C11—C12	1.394 (4)	C26—C27	1.391 (4)
C12—H12	0.9500	C27—H27	0.9500
C12—C13	1.386 (4)	C27—C28	1.383 (4)
C14—H14A	0.9900	C29—H29A	0.9900
C14—H14B	0.9900	C29—H29B	0.9900
			
C15—O2—C14	117.54 (19)	C30—O4—C29	116.43 (19)
H1A—N1—H1B	124 (3)	H2A—N2—H2B	119 (3)
C15—N1—H1A	119 (2)	C30—N2—H2A	118.4 (19)
C15—N1—H1B	116 (2)	C30—N2—H2B	121 (2)
C2—C1—H1	110.4	C17—C16—H16	110.5
C2—C1—C13	102.5 (2)	C28—C16—H16	110.5
C2—C1—C14	110.3 (2)	C28—C16—C17	102.0 (2)
C13—C1—H1	110.4	C29—C16—H16	110.5
C13—C1—C14	112.7 (2)	C29—C16—C17	110.1 (2)
C14—C1—H1	110.4	C29—C16—C28	113.0 (2)
C3—C2—C1	129.3 (3)	C18—C17—C16	129.1 (2)
C3—C2—C7	120.6 (2)	C18—C17—C22	120.4 (2)
C7—C2—C1	110.2 (2)	C22—C17—C16	110.4 (2)
C2—C3—H3	120.7	C17—C18—H18	120.6
C4—C3—C2	118.5 (3)	C17—C18—C19	118.8 (3)
C4—C3—H3	120.7	C19—C18—H18	120.6
C3—C4—H4	119.5	C18—C19—H19	119.5
C3—C4—C5	121.1 (3)	C20—C19—C18	120.9 (3)
C5—C4—H4	119.5	C20—C19—H19	119.5
C4—C5—H5	119.8	C19—C20—H20	119.6
C4—C5—C6	120.4 (3)	C19—C20—C21	120.9 (3)
C6—C5—H5	119.8	C21—C20—H20	119.6
C5—C6—H6	120.6	C20—C21—H21	120.9
C7—C6—C5	118.9 (3)	C22—C21—C20	118.2 (3)
C7—C6—H6	120.6	C22—C21—H21	120.9
C2—C7—C8	108.4 (2)	C17—C22—C23	108.7 (2)
C6—C7—C2	120.5 (2)	C21—C22—C17	120.7 (3)
C6—C7—C8	131.1 (3)	C21—C22—C23	130.6 (3)
C9—C8—C7	130.2 (2)	C24—C23—C22	130.6 (2)
C9—C8—C13	120.7 (2)	C24—C23—C28	120.6 (2)
C13—C8—C7	109.0 (2)	C28—C23—C22	108.7 (2)
C8—C9—H9	120.7	C23—C24—H24	120.7
C10—C9—C8	118.6 (3)	C23—C24—C25	118.6 (3)
C10—C9—H9	120.7	C25—C24—H24	120.7
C9—C10—H10	119.7	C24—C25—H25	119.7
C11—C10—C9	120.7 (3)	C26—C25—C24	120.7 (3)
C11—C10—H10	119.7	C26—C25—H25	119.7
C10—C11—H11	119.6	C25—C26—H26	119.5
C10—C11—C12	120.8 (2)	C25—C26—C27	121.0 (3)
C12—C11—H11	119.6	C27—C26—H26	119.5
C11—C12—H12	120.6	C26—C27—H27	120.6
C13—C12—C11	118.7 (3)	C28—C27—C26	118.8 (3)
C13—C12—H12	120.6	C28—C27—H27	120.6
C8—C13—C1	109.9 (2)	C23—C28—C16	110.2 (2)
C12—C13—C1	129.7 (3)	C27—C28—C16	129.5 (3)
C12—C13—C8	120.4 (3)	C27—C28—C23	120.4 (3)
O2—C14—C1	107.6 (2)	O4—C29—C16	107.33 (19)
O2—C14—H14A	110.2	O4—C29—H29A	110.2
O2—C14—H14B	110.2	O4—C29—H29B	110.2
C1—C14—H14A	110.2	C16—C29—H29A	110.2
C1—C14—H14B	110.2	C16—C29—H29B	110.2
H14A—C14—H14B	108.5	H29A—C29—H29B	108.5
O1—C15—O2	123.1 (2)	O3—C30—O4	123.3 (2)
O1—C15—N1	124.4 (3)	O3—C30—N2	124.2 (3)
N1—C15—O2	112.5 (2)	N2—C30—O4	112.5 (2)
			
C1—C2—C3—C4	179.2 (3)	C16—C17—C18—C19	−179.5 (3)
C1—C2—C7—C6	−178.9 (2)	C16—C17—C22—C21	179.5 (2)
C1—C2—C7—C8	1.5 (3)	C16—C17—C22—C23	−1.2 (3)
C2—C1—C13—C8	2.8 (3)	C17—C16—C28—C23	−2.3 (3)
C2—C1—C13—C12	−178.2 (3)	C17—C16—C28—C27	178.2 (3)
C2—C1—C14—O2	171.9 (2)	C17—C16—C29—O4	−170.1 (2)
C2—C3—C4—C5	−0.4 (4)	C17—C18—C19—C20	0.4 (4)
C2—C7—C8—C9	178.4 (2)	C17—C22—C23—C24	−179.6 (2)
C2—C7—C8—C13	0.4 (3)	C17—C22—C23—C28	−0.3 (3)
C3—C2—C7—C6	0.6 (4)	C18—C17—C22—C21	0.1 (4)
C3—C2—C7—C8	−179.0 (2)	C18—C17—C22—C23	179.4 (2)
C3—C4—C5—C6	0.4 (4)	C18—C19—C20—C21	−0.5 (4)
C4—C5—C6—C7	0.1 (4)	C19—C20—C21—C22	0.3 (4)
C5—C6—C7—C2	−0.6 (4)	C20—C21—C22—C17	−0.1 (4)
C5—C6—C7—C8	179.0 (3)	C20—C21—C22—C23	−179.2 (3)
C6—C7—C8—C9	−1.2 (5)	C21—C22—C23—C24	−0.3 (5)
C6—C7—C8—C13	−179.2 (3)	C21—C22—C23—C28	178.9 (3)
C7—C2—C3—C4	−0.2 (4)	C22—C17—C18—C19	−0.2 (4)
C7—C8—C9—C10	−177.3 (3)	C22—C23—C24—C25	178.0 (3)
C7—C8—C13—C1	−2.1 (3)	C22—C23—C28—C16	1.7 (3)
C7—C8—C13—C12	178.8 (2)	C22—C23—C28—C27	−178.7 (2)
C8—C9—C10—C11	−0.9 (4)	C23—C24—C25—C26	1.1 (4)
C9—C8—C13—C1	179.7 (2)	C24—C23—C28—C16	−179.0 (2)
C9—C8—C13—C12	0.5 (4)	C24—C23—C28—C27	0.6 (4)
C9—C10—C11—C12	0.3 (4)	C24—C25—C26—C27	−0.4 (4)
C10—C11—C12—C13	0.8 (4)	C25—C26—C27—C28	−0.2 (4)
C11—C12—C13—C1	179.9 (3)	C26—C27—C28—C16	179.6 (3)
C11—C12—C13—C8	−1.2 (4)	C26—C27—C28—C23	0.1 (4)
C13—C1—C2—C3	178.0 (2)	C28—C16—C17—C18	−178.6 (3)
C13—C1—C2—C7	−2.6 (3)	C28—C16—C17—C22	2.1 (3)
C13—C1—C14—O2	−74.2 (3)	C28—C16—C29—O4	76.7 (3)
C13—C8—C9—C10	0.5 (4)	C28—C23—C24—C25	−1.2 (4)
C14—O2—C15—O1	−2.3 (4)	C29—O4—C30—O3	6.6 (4)
C14—O2—C15—N1	176.8 (2)	C29—O4—C30—N2	−173.0 (2)
C14—C1—C2—C3	−61.8 (3)	C29—C16—C17—C18	61.2 (3)
C14—C1—C2—C7	117.6 (2)	C29—C16—C17—C22	−118.1 (2)
C14—C1—C13—C8	−115.7 (2)	C29—C16—C28—C23	115.9 (2)
C14—C1—C13—C12	63.3 (4)	C29—C16—C28—C27	−63.7 (4)
C15—O2—C14—C1	150.3 (2)	C30—O4—C29—C16	−158.2 (2)

### (2) Hydrogen-bond geometry (Å, º)

**Table:**

*D*—H···*A*	*D*—H	H···*A*	*D*···*A*	*D*—H···*A*
N1—H1*B*···O3	0.91 (3)	2.06 (3)	2.955 (3)	169 (3)
N1—H1*A*···O1^i^	0.86 (3)	2.08 (3)	2.849 (3)	149 (3)
N2—H2*A*···O1	0.85 (3)	2.13 (3)	2.967 (3)	169 (3)
N2—H2*B*···O3^ii^	0.87 (4)	2.03 (4)	2.827 (3)	152 (3)

Symmetry codes: (i) *x*, *y*−1, *z*; (ii) *x*, *y*+1, *z*.
